# CREB5 promotes invasiveness and metastasis in colorectal cancer by directly activating MET

**DOI:** 10.1186/s13046-020-01673-0

**Published:** 2020-08-25

**Authors:** Shuyang Wang, Junfeng Qiu, Lei Liu, Cailin Su, Lu Qi, Chengmei Huang, Xiaoning Chen, Yaxin Zhang, Yaping Ye, Yanqing Ding, Li Liang, Wenting Liao

**Affiliations:** 1 Department of Pathology, Nanfang Hospital, Southern Medical University, Guangzhou, 510515 Guangdong China; 2 Department of Pathology, School of Basic Medical Sciences, Southern Medical University, Guangzhou, 510515 China; 3 Guangdong Provincial Key Laboratory of Molecular Tumor Pathology, Guangzhou, Guangdong China; 4 Collaborative Innovation Center for Cancer Medicine, Sun Yat-sen University Cancer Center, State Key Laboratory of Oncology in South China, Guangzhou, China

**Keywords:** Colorectal cancer, CREB5, Invasion, Metastasis, MET

## Abstract

**Background:**

cAMP responsive element binding protein 5 (CREB5) is a transcriptional activator in eukaryotic cells that can regulate gene expression. Previously, we found that CREB5 was involved in the occurrence and development of colorectal cancer (CRC) using bioinformatics analysis. However, the biological roles and underlying regulatory mechanism of CREB5 in CRC remain unclear.

**Methods:**

Real-time PCR, western blotting, and immunohistochemistry were used to examine CREB5 expression. In vitro experiments including migration assay, wound-healing assay, chicken chorioallantoic membrane assay, and human umbilical vein endothelial cells tube formation assay were used to investigate the effects of CREB5 on CRC cell migration and tumor angiogenesis ability. Additionally, an orthotopic implantation assay was performed in nude mice to confirm the effects of CREB5 in vivo. Furthermore, gene set enrichment analysis was performed to explore the potential mechanism of CREB5 in CRC.

**Results:**

We found that CREB5 expression was highly upregulated in CRC. CREB5 overexpression was positively correlated with advanced WHO stages and TNM stages and shorter survival in CRC patients. Moreover, CREB5 overexpression promoted while CREB5 silencing reduced the invasiveness and metastatic capacity of CRC cells both in vitro and in vivo. Furthermore, CREB5 directly interacted with the MET promoter and activated the hepatocyte growth factor-MET signalling pathway. Importantly, inhibition of MET reduced the invasion and metastasis of CREB5-overexpressing CRC cells, suggesting that CREB5 promotes metastasis mainly through activation of MET signalling.

**Conclusion:**

Our study demonstrates a crucial role for CREB5 in CRC metastasis by directly upregulating MET expression. CREB5 may be both a potential prognostic marker and a therapeutic target to effectively overcome metastasis in CRC.

## Background

Colorectal cancer (CRC) is one of the most common cancers, ranking third in morbidity and tumor-related mortality among both men and women worldwide. Moreover, approximately 20% of CRC patients have metastases at the time of diagnosis [1]. Metastasis is the leading cause of death among CRC patients [2]. Although systemic treatment of metastatic CRC has improved, the 5-year survival rate is only 12–14%, and because of this poor prognosis, understanding the underlying mechanism of the metastatic process in CRC is critical for both early detection of metastases and more effective treatment [3].

The gene cAMP responsive element binding protein 5 (CREB5), which is located on chromosome 7 (7p15.1), encodes a transcription activator in eukaryotic cells [4]. CREB5 belongs to the ATF/CREB family, the members of which are characterized by a high affinity for cAMP-response elements (CREs) [5]. The targets of the ATF/CREB family include transcriptional regulators (including chromatin-modifying enzymes, coactivators, and corepressors), genes involved in mitochondrial homeostasis and protein import, and genes associated with proliferation and cell cycle entry, metabolism, proteases, transporters, and chaperones [6]. As an ATF/CREB family member, the CREB5 protein contains several important functional domains, including N-terminal zinc finger and C-terminal bZIP domains, the latter of which includes a DNA binding region and leucine zipper [7]. CREB5 is a transcription factor that specifically binds to CRE as a homodimer or a heterodimer with c-Jun or CRE-BP1 and functions as a CRE-dependent trans-activator [8]. CREB5 is physiologically required for embryonic development in mice [9]. Recent studies revealed the roles of CREB5 in the development and progression of cancers. Examination of TCGA pan cancer datasets revealed frequent CREB5 amplification and overexpression in kidney cancers, sarcomas, lymphomas, and lung adenocarcinomas as well as glioblastomas and gliomas [10]. Experimental investigations showed that CREB5 was upregulated in ovarian cancers [11], hepatocellular carcinoma [12], and prostate cancer [10]. High CREB5 expression correlated with a poor prognosis in epithelial ovarian cancer [11] and hepatocellular carcinoma [12]. CREB5 overexpression increased the proliferation of hepatocellular carcinoma [12]. Moreover, overexpression or amplification of CREB5 promoted proliferation and mediated resistance to AR inhibition in metastatic castration-resistant prostate cancers [10]. In silico analysis showed that the CREB5 regulatory network was involved in CRC metastasis [13]. In addition, qRT-PCR assay revealed that CREB5 mRNA was upregulated in CRC tissues and cells [14]. In vitro assays revealed that overexpression of CREB5 resulted in enhanced proliferation and migration and apoptosis inhibition in CRC cells [14]. These findings suggest that CREB5 may play an essential role in the progression of CRC. However, the specific function and molecular mechanism of CREB5 in CRC metastasis remain largely unclear.

Activation of the HGF/MET signalling pathway has been reported to lead to the occurrence and metastasis of a variety of tumors, including CRC, breast cancer, ovarian cancer, lung cancer, and liver cancer [15]. As a tyrosine kinase receptor, MET can be activated by dimerization, multimerization, and phosphorylation after binding to its ligand hepatocyte growth factor (HGF) [16]. Activation of HGF/MET can initiate downstream signalling pathways that drive malignant progression in many types of tumors. MET is considered an essential factor for early invasion and metastasis of CRC and can be regarded as an important prognostic indicator [17, 18]. In the present study, we found that CREB5 promotes CRC invasion and metastasis by increasing MET expression to activate HGF-MET signalling. These results uncover a new molecular mechanism for cancer metastasis and suggest that CREB5 may be a promising target for CRC treatment.

## Materials and methods

### Patients and specimens

A total of 198 pathological specimens were obtained from colon cancer patients between 2009 and 2014 at the Department of Pathology, Nanfang Hospital Southern Medical University. The medical records of these patients provided information on sex, age, and the following essential factors: tumor pathological characteristics, pathologic stage, T stage, Dukes stage, lymph node metastasis, and distant metastasis. Ten pairs of fresh biopsies collected from CRC patients and matched noncancerous mucosal tissue were obtained from the operating room of Nanfang Hospital. The fresh biopsies were stored in liquid nitrogen before use.

In addition, a tissue microarray (No. CO802) containing 78 colon cancer tissue specimens and 2 adjacent noncancerous tissue specimens was purchased from Ailina Biotechnology company. Approval for the use of clinical materials for research purposes was obtained from the Southern Medical University Institutional Board (Guangzhou, China). All samples were collected and analysed with the prior written informed consent of the patients.

### Cell cultures

The human CRC cell lines SW480, HT29, HCT15, HCT116, SW620, LS174t, SW837, LOVO, DLD1 and RKO were purchased from the American Type Culture Collection. SW620, HT29 and LOVO cells were cultured in DMEM medium (Gibco) supplemented with 10% foetal bovine serum (FBS) (Gibco). SW480, HCT116, HCT15, Ls174t, SW837 and DLD1 cells were cultured in RPMI 1640 medium (Gibco) with 10% FBS (Gibco).

### Plasmids

CREB5 constructs were generated by cloning PCR-amplified full-length human CREB5 cDNA into psin-EF2-puro. The following primers were used for cloning (including enzymes): Forward primer, 5′- CGCGAATTCATGATTTATGAGGAATCCAA-3′ (EcoR I); reverse primer, 5′-CCGGCTAGCTTAAAGAATCGGATTCAGGT-3′ (Nhe I). For deletion of CREB5, 2 short hairpin RNA (shRNA) sequences (CREB5 shRNA1: 5′-AACAAGTCATCCAGCATAA-3′; CREB5 shRNA2: 5′-GGAATATCTCGATGCATAA-3′) were separately cloned into a GV248 vector. Psin-EF2-puro and GV248 were purchased from Addgene Inc.

### RNA isolation, reverse transcription (RT) and real-time PCR

Total RNA samples from cultured cells and primary tumor tissues were extracted using Trizol reagent (Invitrogen, USA) according to the manufacturer’s instruction. Real-time RT-PCR was performed at least three times in triplicate using SYBR GREEN MIX (TOYOBO, Japan) and the ABI PRISM 7500 Sequence Detection System (Applied Biosystems, USA). The data were normalized to the geometric mean of the housekeeping gene GAPDH and calculated using the 2^-ΔΔCT^ method. Primer Express was used to design the real-time PCR primers, and primer sequences for amplification are shown in Supplementary Table S2.

### Western blotting analysis

We carried out western blotting as previously described [19] using anti-CREB5 (Abcam, ab168928), anti-MET (Cell Signaling Technology, #8198), anti-p-MET(Cell Signaling Technology, #3077), anti-AKT (Cell Signaling Technology, #2920), anti-p-AKT(Cell Signaling Technology, #13038), anti-ERK(Cell Signaling Technology, #4695), anti-p-ERK (Cell Signaling Technology, #4370), and anti-Snail (Cell Signaling Technology, #3879) antibodies. Mouse monoclonal anti-α-tubulin antibody (Sigma, #9026) was used as the internal control.

### Immunohistochemistry

Immunohistochemistry (IHC) staining was performed as previously described using CREB5 antibody (Abcam, USA, ab168928) [20]. The degree of IHC staining was reviewed and scored independently by two observers based on both the proportion of positively stained tumor cells and the intensity of staining [18, 19]. The proportion of tumor cells was scored as follows: 0 (no positive tumor cells), 1 (< 10% positive tumor cells), 2 (10–50% positive tumor cells), and 3 (> 50% positive tumor cells). The intensity of staining was graded according to the following criteria: 0 (no staining); 1 (weak staining = light yellow), 2 (moderate staining = yellow brown), and 3 (strong staining = brown). The staining index was calculated as the staining intensity score × the proportion of positive tumor cells. Using this method of assessment, we evaluated the expression of CREB5 in benign breast epithelium and malignant lesions by determining the staining index, with scores of 0, 1, 2, 3, 4, 6, and 9. Cutoff values for CREB5 were selected on the basis of a measure of heterogeneity with the log-rank statistical test with respect to overall survival. Optimal cutoff values were identified: a staining index ≥4 was used to define tumors with high CREB5 expression, and an index ≤3 was used to define tumors with low CREB5 expression.

### Luciferase reporter assay

Genomic DNA extracted from SW480 cells was used as a template to amplify MET promoter fragments. MET promoter fragments were obtained by PCR and constructed into pGL3-Basic plasmid using a fast ligation kit following manufacturer’s instructions (Sangon, B620511–0100). The sequences of the PCR primers are listed in Supplementary Table S4. Cells at 60% confluence in a 24-well plate were transfected using Lipofectamine 2000. Forty-eight hours after transfection, luciferase activity was measured using the dual-luciferase reporter assay system (Promega corp., Madison, WI, USA) and normalized to Renilla luciferase gene expression. All the experiments were performed in triplicate.

### Chromatin immunoprecipitation (ChIP) assay

ChIP assays were carried out as previously described [20]. Precleared lysates were incubated with CREB5 antibody (Abcam, ab168928) or normal mouse immunoglobulin G (CST #5946) as a negative control overnight at 4 °C with rotation. The human MET promoter was amplified by PCR. All ChIP assays were performed three times, and the sequences of the PCR primers are listed in Supplementary Table S3.

### Migration assay

A Boyden chamber with an 8-μm-pore filter membrane was used for the in vitro migration and invasion assay. Briefly, cells (5 × 10^4^) in culture medium containing 1% FBS were seeded in the upper chamber, and culture medium with 10% FBS was added in the lower chamber as a chemoattractant. The upper side of the filter was first coated with 0.2% Matrigel (BD Biosciences, San Jose, CA, USA). After incubation for 24 h, cells on the upper side of the filter were removed with cotton swabs. Cells that migrated to the lower surface of the filter were fixed in 4% paraformaldehyde and stained with Giemsa. The migratory cells were counted (10 random 200× fields per well). Three independent experiments were performed, and the data are presented as the mean ± s.e.m.

### Wound-healing assay

Cells were seeded in 6-well plates and incubated under permissive conditions until 90% confluence. After serum starvation for 24 h, wounds were created in the confluent cells using a pipette tip. Wound healing within the scrape line was then observed and photographed at indicated time points. Each experiment was repeated at least three times.

### Chicken chorioallantoic membrane assay

A chicken chorioallantoic membrane (CAM) assay was performed on the sixth day of development of fertilized chicken eggs as previously described [21].

### Human umbilical vein endothelial cell tube formation assay

First, 200 μL of Matrigel was pipetted into each well of 24-well plates and polymerized for 30 min at 37 °C. Human umbilical vein endothelial cells (HUVECs; 5 × 10^4^) in 200 μL of conditional medium were added to each well and incubated at 37 °C in 5% CO_2_ for 8 h. Images were obtained under a bright-field microscope, and the capillary tubes were quantified by the counting length.

### Orthotopic mouse metastatic model

4- to 6-week-old Balb/C athymic nude mice (nu/nu) were obtained from the Animal Center of Southern Medical University, Guangzhou, China. All mice were housed in a sterile environment. Cells (2 × 10^6^ per mouse) were orthotopically inoculated into the caecum of anaesthetized nude mice. The mice were sacrificed within 10 weeks after surgery, individual organs were excised, and metastases were observed by histological analysis. Tissues were then fixed with formaldehyde and paraffin-embedded, and then 5-mm sections were cut and stained with haematoxylin and eosin (H&E). The numbers of gross metastatic foci were determined using a dissection microscope. All the mice used in this study were maintained under specific pathogen-free conditions, and all animal experiments were conducted in accordance with standard procedures and approved by the Institutional Use Committee for Animal Care.

### Statistical analysis

All statistical analyses were carried out using SPSS version 20.0. Pearson correlation analysis was used for expression correlation analysis. The survival curves of CRC patients in low- and high-CREB5 expression groups were analysed by the Kaplan-Meier method, and the log-rank test was used to compare differences. *P* < 0.05 was considered significant.

## Results

### CREB5 is upregulated in CRC and associated with a poor prognosis

**Fig. 1:**
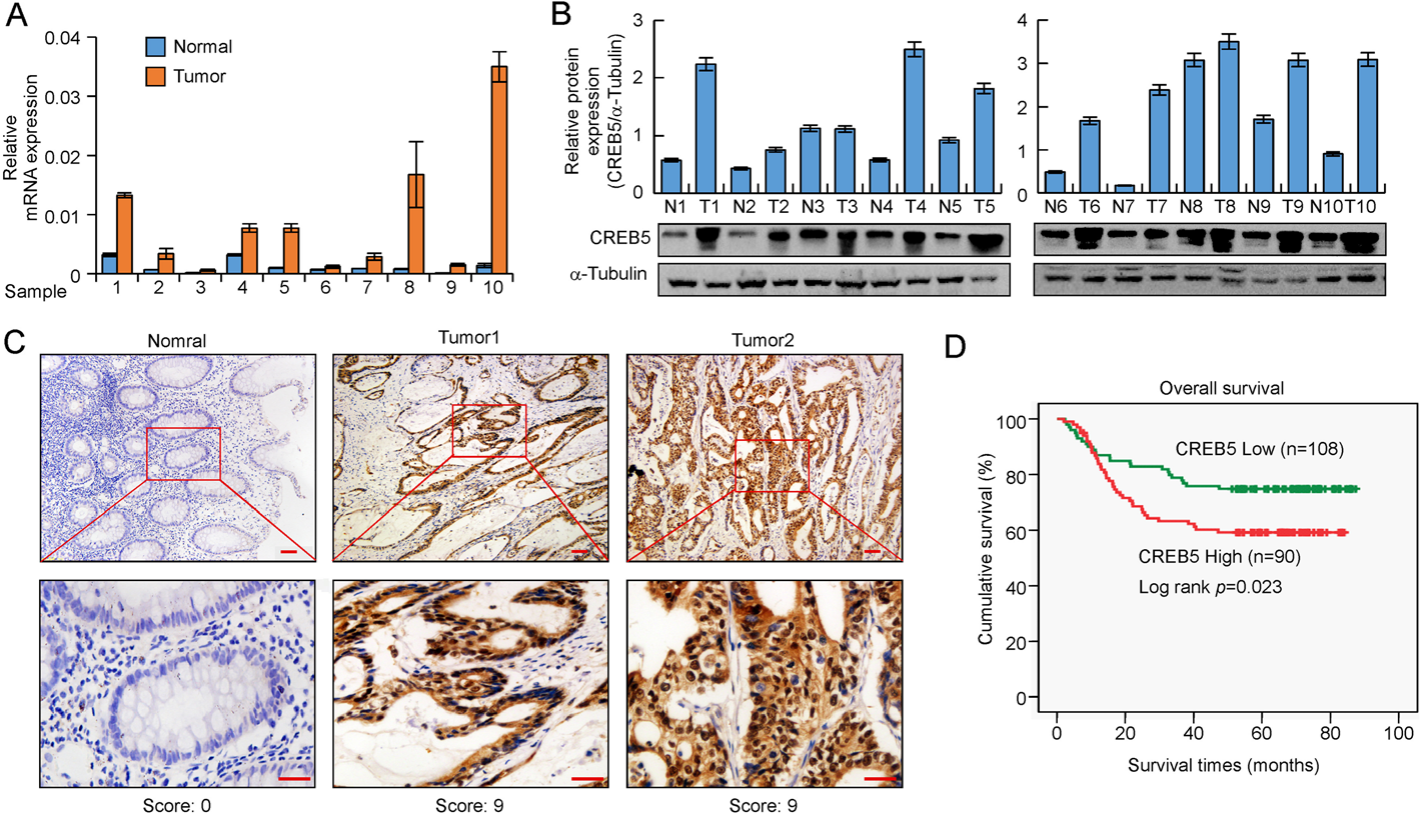
CREB5 is upregulated in CRC and associated with a poor prognosis. **a** and **b** Real-time PCR and western blotting analysis of CREB5 expression in paired human colon cancer tissues and adjacent noncancerous tissues (*p* < 0.01). Quantity One software was used to quantify the protein expression levels. **c** IHC representative images of CREB5 expression in normal intestinal epithelium and CRC tissues. Scale bar: 50 μm. **d** The paraffin samples of 198 CRC patients were divided into a low-CREB5 expression group (*n* = 108) and a high-CREB5 expression group (*n* = 90) based on IHC results. The Kaplan-Meier method was used to analyse survival curves, and the log-rank test was used to compare differences (*p* = 0.023)

The expression of CREB5 was analysed in 20 different types of malignant tumors in the public database Oncomine (www.conomine.com) [22], revealing that CREB5 was upregulated in CRC tissues in 9 of 17 CRC datasets (Supplementary Fig. S1A). Additionally, a gene set enrichment analysis (GSEA) plot showed significant enrichment of tumor-related genes and CRC-related gene sets in the high-CREB5 expression group (Supplementary Fig. S1B). Real-time PCR and western blotting analyses showed that CREB5 was differentially expressed in CRC cell lines (Supplementary Fig. S1C-D). In addition, CREB5 was significantly upregulated in ten CRC tissues compared with adjacent normal intestinal epithelial tissues (Fig. 1a and b). IHC showed that CREB5 protein was weakly expressed in normal tissue but markedly increased in adenocarcinoma cells and was mainly localized in the nuclei (Fig. 1c). Kaplan-Meier survival analysis showed that CRC patients with higher CREB5 protein expression levels had a poorer prognosis (Fig. 1d). In addition, CREB5 expression levels were significantly correlated with the T classification, lymph node metastasis, and distant metastasis (*p* < 0.001, Supplementary Table S1). CREB5 expression was substantially higher in tumors from patients with distant metastasis. Moreover, high CREB5 expression was also positively correlated with WHO stages (*p* < 0.001, Supplementary Table S1). These data suggested that CREB5 expression is significantly correlated with advanced stages of CRC.

### CREB5 activates MET signalling

**Fig. 2:**
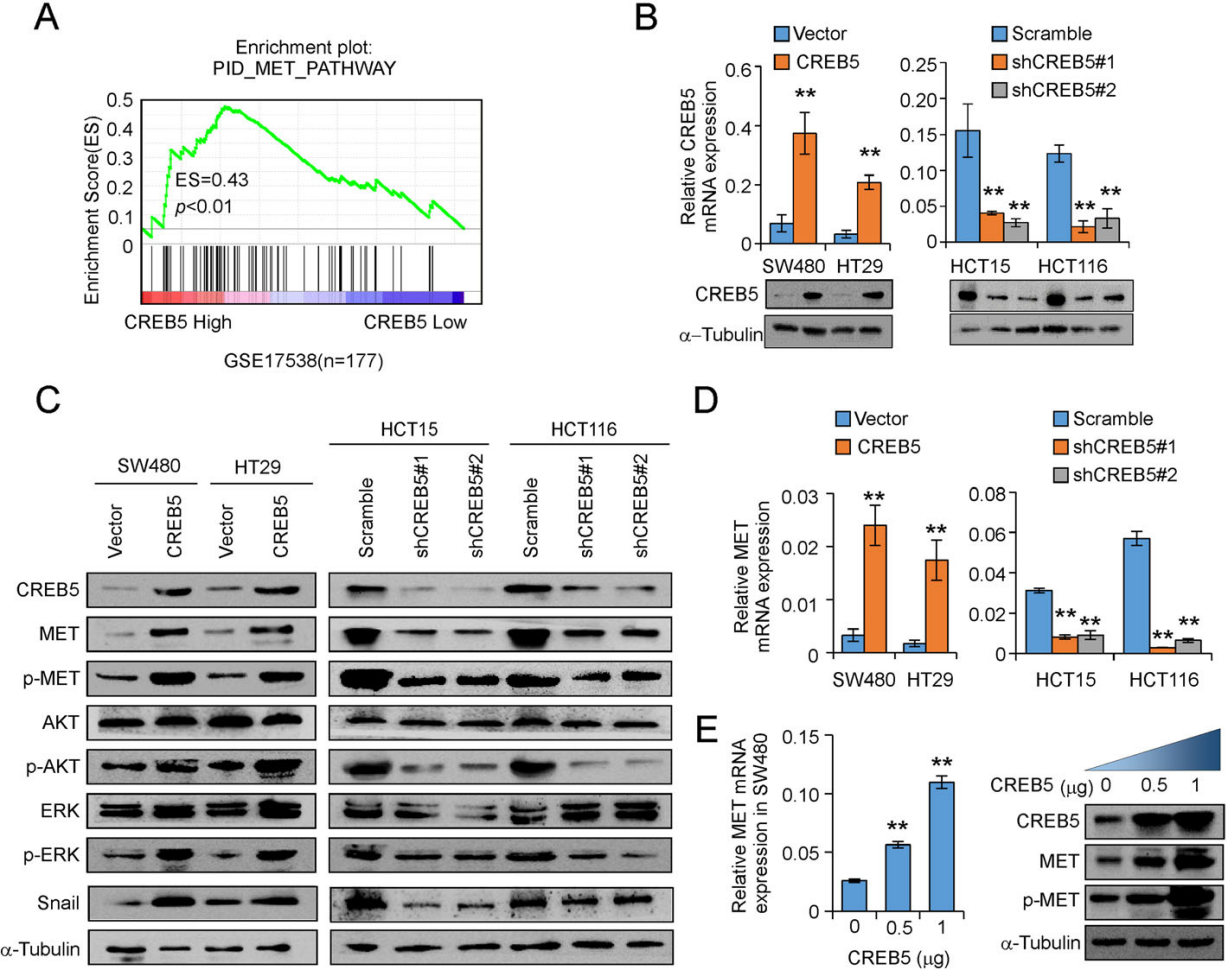
CREB5 activates the MET signalling pathway. **a** GSEA of GSE17538 in MET signalling pathways (ES = 0.43, *p* < 0.01). **b** Stable overexpression and interference cell lines were detected by western blotting and real-time PCR. **c** The expression of MET and downstream signalling molecules in CREB5-knockdown or CREB5-overexpressing cells was observed by western blotting. **d** CREB5 had an effect on MET by real-time PCR in the indicated cells. **e** After transient transfection of different amounts of the CREB5-overexpression plasmid in SW480 cells, the protein and mRNA levels of MET were detected by western blotting and real-time PCR, respectively. ** *p* < 0.01

GSEAs of CREB5-regulated gene signatures revealed that higher CREB5 expression was positively correlated with enrichment of an MET signalling pathway signature (GSE17538; Fig. 2a). To validate this result, we established stable CREB5-overexpressing and CREB5-knockdown CRC cell lines (Fig. 2b). Upregulation of CREB5 significantly increased while knockdown of CREB5 decreased the expression of total MET at both translational and transcriptional levels (Fig. 2c and d). In addition, CREB5 overexpression markedly increased but CREB5 downregulation significantly attenuated the expression of phosphorylated MET, p-ERK, p-AKT, and Snail (Fig. 2c). Moreover, MET expression was increased in a dose-dependent manner at both translational and transcriptional levels by transiently transfecting SW480 cells with a CREB5 expression vector (Fig. 2e).

### CREB5 associates with the MET promoter

**Fig. 3:**
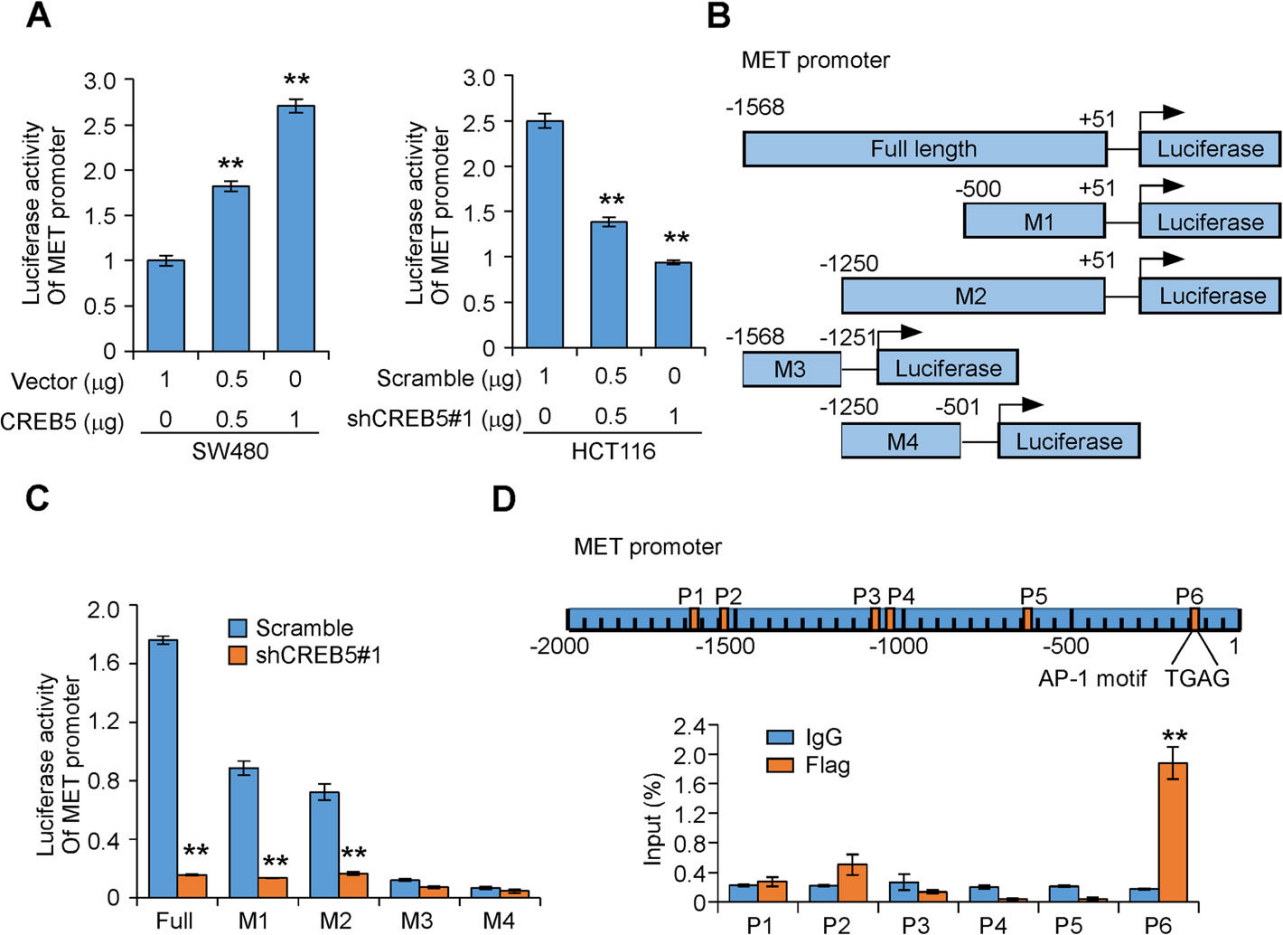
CREB5 regulates MET and binds directly to the MET promoter. **a** The MET promoter sequence was cloned into pGL3-Basic vector containing the luciferase reporter gene and then transfected into CRC cells with the indicated treatments. **b** Schematic diagram of the full and truncated MET promoter. **c** The full-length MET promoter or its truncations were cloned into pGL3-Basic vector containing the luciferase reporter gene and then transfected into HCT116 cells with CREB5 shRNA or empty vector. **d** ChIP analysis of CREB5 binding to the MET promoter in SW480 cells. ** *p* < 0.01

Given that CREB5 is a transcriptional factor and upregulates MET at the transcriptional level, we performed a luciferase reporter assay to investigate whether CREB5 can increase MET promoter activity. A 2.7-kb fragment of the full-length MET promoter region was subcloned into a luciferase vector [20]. MET promoter activity was increased by co-transfection with a CREB5 expression vector in SW480 cells but decreased in HCT116 cells expressing CREB5 shRNA in a dose-dependent manner compared with empty vectors (Fig. 3a). To determine the effective regions of the MET promoter that CREB5 may affect, we transfected MET promoter truncations (Fig. 3b) into HCT116 cells expressing either CREB5 shRNA or a Scramble control sequence. As shown in Fig. 3c, luciferase activity was increased in cells carrying the full-length MET promoter and truncations − 500 and − 1250 bp upstream of the transcription start site but not in cells carrying truncations − 1251 to − 1568 bp or − 501 to − 1250 bp. Knockdown of CREB5 expression by co-transfection of CREB5 shRNA significantly decreased MET promoter activity (Fig. 3c). Furthermore, we performed chromatin immunoprecipitation (ChIP) assays and identified that the − 68 to − 223-bp region of the MET promoter, which contains an AP-1 motif, was a CREB5 protein binding site (Fig. 3d). These data identify MET as a direct transcriptional target of CREB5.

### Downregulation of CREB5 represses invasiveness and reduces the metastatic potential of CRC cells

**Fig. 4:**
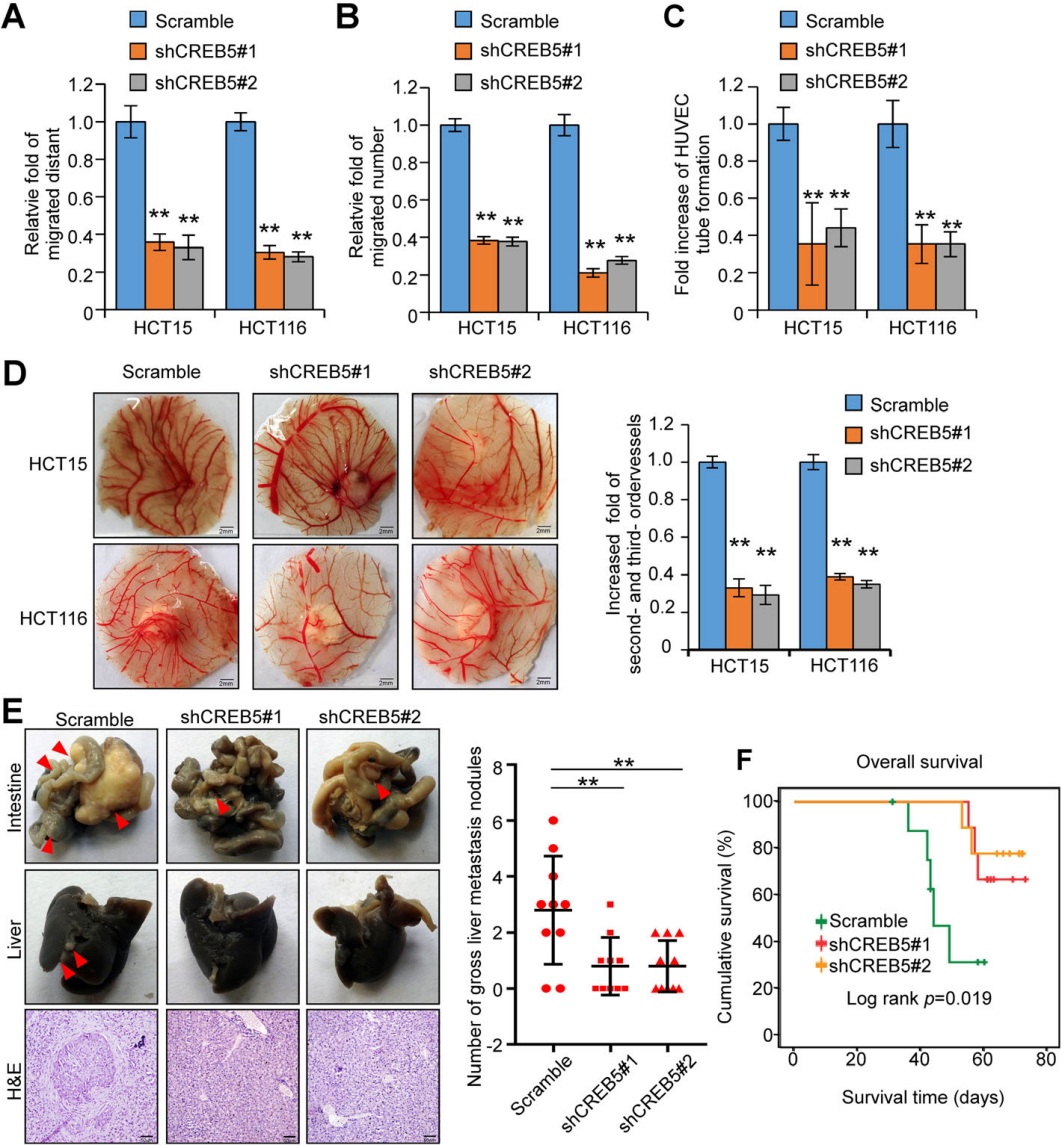
Downregulation of CREB5 inhibits the invasion and metastasis of CRC cells in vivo and in vitro. **a** and **b** Wound-healing assay and transwell migration assay were performed to evaluate the invasive and migratory abilities of CRC cells with different treatments in vitro. **c** HUVEC tube formation after stimulation with the indicated conditioned medium. **d** Representative images of the CAM assay. Histograms show the formation of secondary and tertiary blood vessels after stimulation with the indicated conditional medium. Scale bar: 2 mm. **e** Orthotopic transplantation with the indicated HCT116 cells in nude mice (*n* = 9 in each group) was performed, and representative gross images of the livers and intestines are shown. The arrows indicated the tumors. Liver sections were stained with haematoxylin and eosin (H&E). Scale bar: 50 μm. **f** The Kaplan-Meier method was used to analyse survival curves in the specified treatment groups, and the log-rank test was used to compare differences. ** *p* < 0.01

Next, we investigated the role of CREB5 in invasiveness and metastasis in CRC cells. Silencing of CREB5 significantly compromised the migratory and invasive abilities of CRC cells (Fig. 4a and b, Supplementary Fig. S2A and B). The tubule formation and chicken CAM assays revealed that knockdown of CREB5 strongly inhibited the formation of tubules by HUVECs and inhibited angiogenesis in CAMs (Fig. 4c and d, Supplementary Fig. S2C). Orthotopic inoculation assay showed that knockdown of CREB5 inhibited liver metastases (Fig. 4e). Knockdown of CREB5 also obviously extended the overall survival time of nude mice inoculated with the CRC cell lines (Fig. 4f). These results indicate that silencing CREB5 inhibits the invasiveness and metastasis of CRC cells.

### Inhibition of MET attenuates the invasion and metastasis of CRC cells by CREB5 in vivo and in vitro

**Fig. 5:**
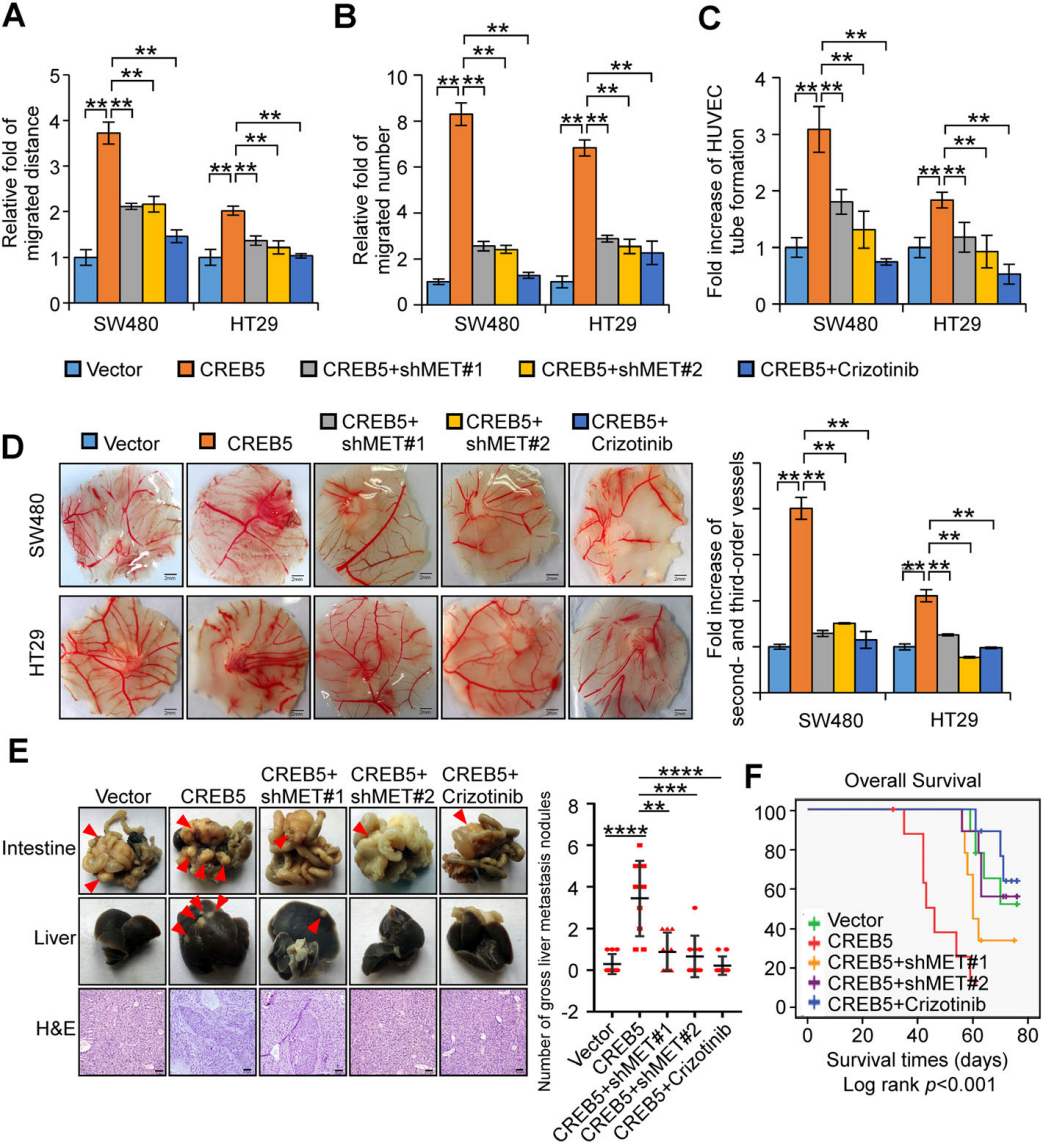
Overexpression of CREB5 promotes the invasion and metastasis of CRC cells, but inhibition of MET weakens these effects. **a** and **b** The invasive and migratory abilities of CRC cells in vitro with different treatments were evaluated by wound-healing assay and transwell migration assay. **c** HUVEC tube formation after stimulation with the indicated conditional medium. **d** Representative images of the CAM assay. Histograms show the formation of secondary and tertiary blood vessels after stimulation with the indicated conditional medium. Scale bar: 2 mm. **e** Orthotopic transplantation with the indicated SW480 cells in nude mice (n = 9 in each group) was conducted, and representative gross images of the livers and intestines are shown. The arrows indicate the tumors. Liver sections were stained by H&E. Scale bar: 50 μm. **f** The Kaplan-Meier method was used to analyse the survival curves of different treatment groups, and the log-rank test was used to compare differences. ** *p* < 0.01, *** *p* < 0.001, **** *p* < 0.0001

To determine the functional relationship between CREB5 and MET in the invasion and metastasis of CRC, we knocked down MET using two MET shRNAs or suppressed MET activation using the MET inhibitor crizotinib (EMD 1214063). Silencing or inhibition of MET in SW480/CREB5 or HT29/CREB5 cells significantly decreased the expression of phosphorylated MET, p-ERK, and p-AKT as well as Snail (Supplementary Fig. S3A). In addition, the invasive and migratory abilities of SW480/CREB5 or HT29/CREB5 cells were partially diminished by inhibition of MET (Fig. 5a and b, Supplementary Fig. S3B and C). Moreover, upregulation of CREB5 expression enhanced the capacity of CRC cells to induce tube formation and angiogenesis in CAMs. In contrast, angiogenesis ability was partially diminished by MET inhibition (Fig. 5c and d, Supplementary Fig. S3D). Orthotopic inoculation assay showed that CREB5 significantly promoted liver metastases and decreased the overall survival of mice (Fig. 5e and f). Conversely, inhibition of MET significantly attenuated the formation of metastatic foci by SW480/CREB5 cells and extended the survival time of mice inoculated with SW480/CREB5 cells (Fig. 5e and f).

### CREB5 expression positively correlates with MET expression in CRC

**Fig. 6:**
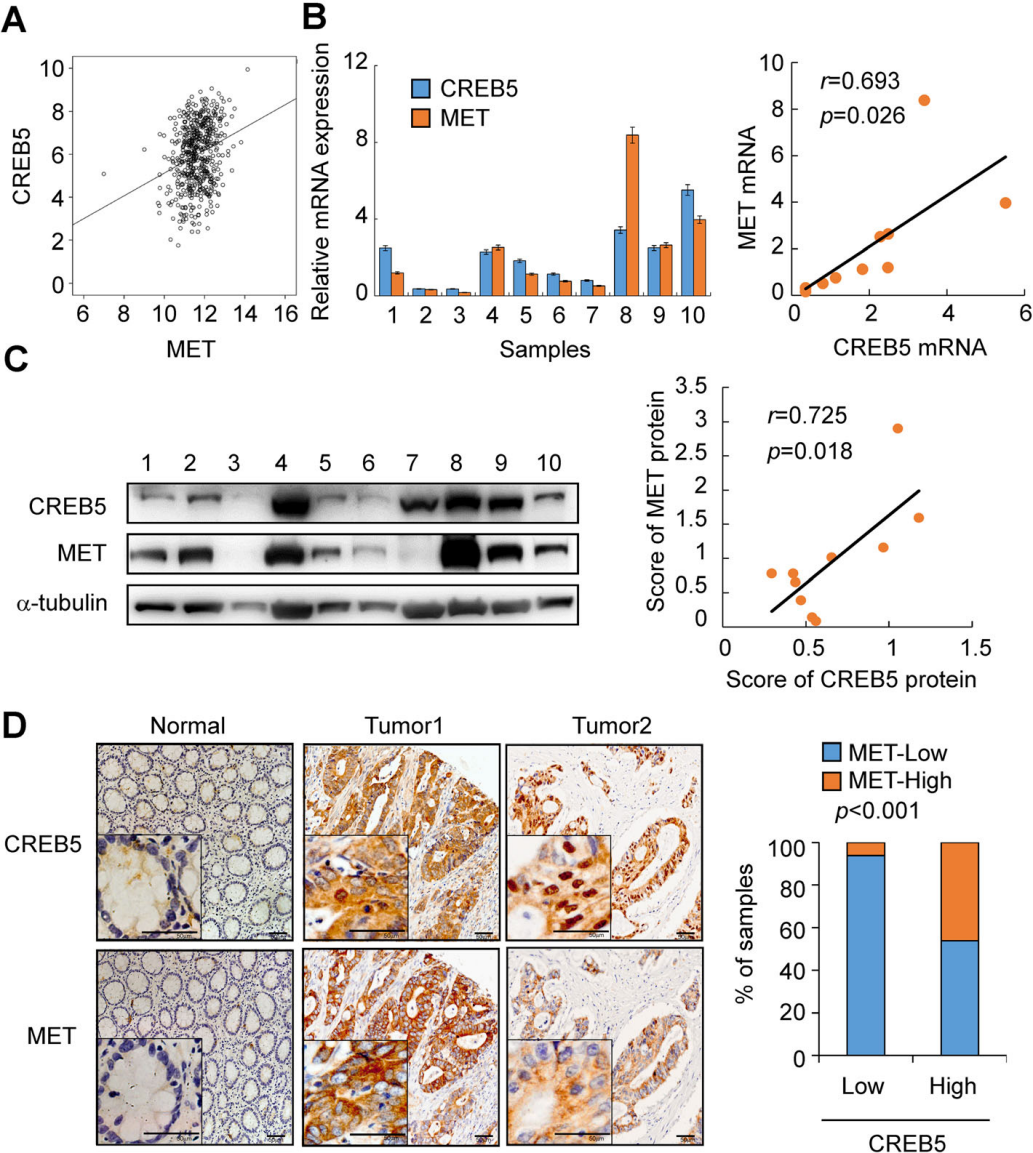
CREB5 positively correlated with MET expression in CRC. **a** Correlation analysis of CREB5 and MET in TCGA CRC data (*r* = 0.260, *p* < 0.001). **b** and **c** Correlation analysis of CREB5 and MET at the mRNA (*r* = 0.693, *p* = 0.026) and protein levels (*r* = 0.725, *p* = 0.018) in 10 fresh CRC tissues. **d** The expression of CREB5 and MET protein in specimens, including 78 colon cancer tissue specimens and 2 adjacent normal tissue specimens, was detected by IHC. Representative IHC images (left) and correlation analysis (right) of CREB5 and MET expression. Scale bar: 50 μm. High expression of CREB5: *n* = 73 (high expression of MET: *n* = 52). Low expression of CREB5: n = 5 (low expression of MET: *n* = 4) (*p* < 0.001)

To assess a potential link between CREB5 and MET expression in human CRC, we analysed TCGA CRC data and identified a strong positive correlation between high expression levels of CREB5 and MET (*p* < 0.001, *r* = 0.26, Fig. 6a). In addition, analyses of 10 fresh CRC tissues showed that CREB5 expression was positively correlated with MET expression at both mRNA (*p* = 0.026, *r* = 0.693) and protein levels (*p* = 0.018, *r* = 0.725) (Fig. 6b and c). Furthermore, IHC revealed that CREB5 expression was positively correlated with MET (Fig. 6d, *p* < 0.001).

## Discussion

Metastasis of CRC is a multistep process requiring the accumulation of genetic and/or epigenetic alterations and abnormal expression of genes involved in signal transduction pathways, including oncogenic mutation of KRAS and activation of the ERK/MAPK pathway, Wnt/β-Catenin signalling and TGFβ signalling [23]. AP-1 DNA binding sequences act as crucial response elements for transcriptional activation by the RAS/ERK pathway [24]. CREB5 is a CRE-dependent trans-activator downstream of the RAS/ERK signalling pathway since it interacts with c-Jun, which is one of the AP-1 subunits, to form a homodimer or a heterodimer [8]. Along with FOXD1 and ATF3, CREB5 formed a transcription factor regulatory network that negatively regulates MAPK signalling, which is suppressed by FZD3 in melanoma [25]. Previous studies have revealed that CREB5 mRNA was upregulated in CRC as demonstrated by bioinformatics analysis or qRT-PCR examination in human cancer tissues [13, 14]. In the current study, we demonstrated that CREB5 was highly upregulated in CRC at both the mRNA and protein levels. Overexpression of CREB5 was significantly associated with aggressive cellular characteristics of CRC (e.g., an advanced WHO stage and an advanced TNM stage) and poorer patient outcomes, suggesting that CREB5 might be an oncogene and a prognostic marker of CRC progression. Consistent with our results, upregulation of CREB5 has been reported to be responsible for poorer outcomes in patients with epithelial ovarian cancer [11] and hepatocellular carcinoma [12].

In prostate cancers, CREB5 overexpression occurs through both copy number gain and increased gene expression [10]. However, examination of TCGA colorectal cancer datasets via cBioPortal revealed rare amplification of CREB5, suggesting that overexpression of CREB5 is controlled at transcriptional and post-transcriptional levels. CREB5 has been shown to be a downstream target of LncRNA SNHG5/miR-132-3p [14] and circular RNA circVAPA/miR-125a [26]. In addition, FZD3 inhibits transcriptional networks controlled by CREB5 [25]. However, the alternative mechanisms involved in upregulation of the CREB5 gene and activation of CREB5-mediated signalling require further investigation.

The effects of CREB5 overexpression on promoting cell proliferation and migration have been demonstrated using in vitro assays in human hepatocellular carcinoma cells and CRC cell lines [12, 14]. In the current study, we showed that CREB5 overexpression promoted while CREB5 silencing reduced the invasiveness and metastatic capacity of CRC cells both in vitro and in vivo. Mechanistically, CREB5 directly interacted with the MET promoter and activated the HGF-MET signalling pathway. Importantly, inhibition of MET reduced the invasion and metastasis of CREB5-overexpressing CRC cells, suggesting that CREB5 promotes metastasis mainly through activation of MET signalling. Our data provide solid evidence that upregulation of CREB5 plays an essential role in CRC metastasis. Recently, overexpression or amplification of CREB5 was reported to promote proliferation and mediate resistance to AR inhibition in metastatic castration-resistant prostate cancers [10]. These data suggest that CREB5 may function as a multi-tasking regulator in cancer progression and clinical outcomes.

CREB family members can be phosphorylated via various intracellular signal transduction pathways, such as protein kinase A (PKA), calmodulin-dependent protein kinase (CaMK), mitogen-activated protein kinases (MAPKs), and other kinases [27]. Upon phosphorylation, CREB recruits CREB-binding protein (CBP) and binds to the CREs of the promoters of its target genes [28]. Target genes containing consensus sites for CREB binding include those related to metabolism, transcription, neuropeptides/neurotransmitters, cell cycle/cell survival/DNA repair, growth factors, immune regulation, reproduction/development, signalling, transport, and structure [29]. Specifically, knockdown of CREB1/CREB5 increased tumor necrosis factor alpha (TNF-α) levels, enhanced the expression of phospho-NF-κB p65 and NF-κB p65, and induced immunosuppression in monocytes [30]. In prostate cancer, CREB5 could improve resistance to enzalutamide with the help of FOXA1 and selectively enhance the interaction of AR with target genes critical for survival [10]. However, little is known about the downstream targets of CREB5 involved in the progression of CRC. Our results showed that CREB5 directly interacted with the MET promoter and activated the HGF/MET signalling pathway, which in turn increased the expression of downstream ERK and PI3K signalling cascades. Meanwhile, the expression of Snail, an essential EMT transcription factor, was also upregulated via the CREB5/HGF/MET axis.

The HGF/MET signalling pathway is one of the major causes of tumor cell migration [31]. The tyrosine kinase MET is activated following interaction with the HGF ligand and transmits intracellular signals via the MAPK and PI3K/AKT signalling cascades [16]. Activation of the HGF-MET signalling pathway promotes metastasis of various tumors, including CRC, breast cancer, ovarian cancer, lung cancer and liver cancer [32–35]. In most cases, MET is activated by gene amplification, mutation, overexpression and alternative splicing [36, 37]. However, MET gene amplification is a rare event in CRC. Transcriptional regulation may be one of the major mechanisms controlling MET expression. MET has been identified to be a target of hypoxia inducible factor-1 (HIF1), FOXC2, and MACC1 in CRC [20, 38, 39]. In the present study, we demonstrated that CREB5 is a novel transcriptional factor that interacts with the MET promoter at the AP-1 motif and activates MET expression.

## Conclusion

In conclusion, our data suggest that CREB5 has an essential role in CRC metastasis by regulating the proto-oncogene MET. Interfering with CREB5 may represent an alternative therapeutic target to prevent or reduce metastasis in CRC.

## Supplementary information

**Additional file 1: Table S1.** The relationship between CREB5 expression and clinicopathological parameters. **Table S2.** Primer sequences used for real-time PCR (5′ to 3′). **Table S3.** Sequence of primers for ChIP assay. **Table S4.** Sequence of primers for Luciferase reporter assay.

**Additional file 2: Figure S1.** Bioinformatics analysis of CREB5 expression. The expression of CREB5 in CRC and other malignant tumors was analyzed by Oncomine database. The inclusion criteria were that the difference of CREB5 expression between tumor tissue and normal tissue was more than 2 times, and the arrangement of gene position was less than 10% with *P* < 0.001. The outliers in the red and blue boxes represent the number of data sets with high and low expression of CREB5, respectively. The right table of (A) represents the COPA score of CREB5 in 17 CRC data sets. (B) The two CRC chips (GSE17538, *n* = 177, and GSE35896, *n* = 66) from the public database of GEO was analyzed by GSEA. The plot showed significant enrichment of tumor-related gene set (KEGG_PATHWAY_IN_CANCER) and colorectal cancer-related gene set (KEGG_COLORECTAL_CANCER) in the CREB5 high expression group. (C and D) Real-time PCR and western blotting analysis of CREB5 endogenous expression in indicated CRC cells.

**Additional file 3: Figure S2.** Representative images of wound-healing assay (A), transwell migration assay (B) and HUVEC tube formation assay (C) with indicated treatment. Scale bars, 100 μm in (A) and (C). 50 μm in (B).

**Additional file 4: Figure S3.** Two MET shRNAs or inhibitors (Crizotinib) were used in SW480 and HT29 cells overexpressed CREB5, and the expression of MET, p-MET, p-AKT and p-ERK were analyzed by western blotting. ** *p* < 0.01. Representative images of wound-healing assay (B), transwell migration assay (C) and HUVEC tube formation assay (D) with indicated treatment were shown. Scale bars, 100 μm in (B) and (D). Scale bars, 50 μm in (C).

## Data Availability

All data generated or analysed during this study are included in this published article and its supplementary information files.

## Abbreviations

CREB5: cAMP responsive element binding protein 5
CRC: Colorectal cancer
CAM: Chicken chorioallantoic membrane
HUVEC: Human umbilical vein endothelial cells
GSEA: Gene set enrichment analysis
HGF: Hepatocyte growth factor
CREs: cAMP-response elements
FBS: Foetal bovine serum
shRNA: Short hairpin RNA
IHC: Immunohistochemistry
SI: Staining index
H&E: Haematoxylin and eosin
ChIP: Chromatin immunoprecipitation
