# Supplementary material for: CREB5 promotes invasiveness and metastasis in colorectal cancer by directly activating MET
Source: J Exp Clin Cancer Res. 2020 Aug 25;39:168. doi: 10.1186/s13046-020-01673-0 (PMC7446182; doi:10.1186/s13046-020-01673-0)
Supplement: Supplementary file 1 — Additional file 1: Table S1. The relationship between CREB5 expression and clinicopathological parameters. Table S2. Primer sequences used for real-time PCR (5′ to 3′). Table S3. Sequence of primers for ChIP assay. Table S4. Sequence of primers for Luciferase reporter assay. [file 13046_2020_1673_MOESM1_ESM.docx]

**Supplementary Tables**

**Supplementary Table 1. The relationship between CREB5 expression and clinicopathological parameters.**

The clinicopathological data of 198 CRC patients were collected, such as age, gender, histology, differentiation, dukes’ stage and TNM stage. The relationship between CREB5 expression and clinicopathological parameters was analyzed using IPP software and semi-quantitative methods.

| **Characteristics** | **CREB5 levels** | | **P-value** |
| --- | --- | --- | --- |
|  | **Low** | **High** |  |
| **Age** |  |  |  |
| ≤mean(57) | 53 | 47 | 0.877 |
| ＞mean(57) | 55 | 43 |  |
| **Gender** |  |  |  |
| Male | 62 | 48 | 0.776 |
| Female | 46 | 42 |  |
| **Histology** |  |  |  |
| Columnar adenocarcinoma | 81 | 75 | 0.320 |
| Mucinous adenocarcinoma | 13 | 6 |  |
| Others | 14 | 9 |  |
| **Differentiation** |  |  |  |
| Well and moderate | 85 | 76 | 0.361 |
| Poor and undifferentiated | 23 | 14 |  |
| **WHO stage** |  |  |  |
| Stage Ⅰ | 20 | 8 | ＜0.001 |
| Stage Ⅱ | 59 | 17 |  |
| Stage Ⅲ | 17 | 32 |  |
| Stage Ⅳ | 12 | 33 |  |
| **T stage** |  |  |  |
| 1 | 1 | 10 | 0.004 |
| 2 | 21 | 0 |  |
| 3 | 67 | 44 |  |
| 4 | 19 | 36 |  |
| **Lymph node involvement** |  |  |  |
| No | 81 | 33 | ＜0.001 |
| Yes | 27 | 57 |  |
| **Distant metastasis** |  |  |  |
| No | 96 | 57 | ＜0.001 |
| Yes | 12 | 33 |  |

**Supplementary Table S2. Primer sequences used for real-time PCR (5' to 3').**

| **Gene** | **Sense** | **Anti-sense** |
| --- | --- | --- |
| CREB5 | CCCTGCCCAACCCTACAATG | GGACCTTGCATCCCCATGAT |
| MET | ATTGTAGAGCTTCGGCAGTTATC | CTGTAAACACCAACTCATTGCG |
| GAPDH | ACAGTCAGCCGCATCTTCTT | GACAAGCTTCCCGTTCTCAG |

**Supplementary Table S3. Sequence of primers for ChIP assay.**

| **MET-chip-primer** | **Sequence (5’ to 3’)** |
| --- | --- |
| MET -1-left | TCACTCAGCCTCCATAGTTA |
| MET -1-right | GGCTTGCAAAATGTGTTTTC |
| MET -2-left | AATGTTTCCCAGGAGTTTGT |
| MET -2-right | TCATGTTGTACTACCAGCAC |
| MET -3-left | CTTCTACTGGGTATCAGTGC |
| MET -3-right | ATTCAAAAGTTGTCACCCAC |
| MET -4-left | TACAGAATATCTAAGTATTTATTGA |
| MET -4-right | ATTTCCAAACAAGGATGGT |
| MET -5-left | CTAAACTGAAGGTACAGGAA |
| MET -5-right | GGAACTCAATAAATACTTAGATA |
| MET -6-left | ATATTAACGCGTGGGCGGAGGG |
| MET -6-right | GCGAGATCTGCCCTCCACTCG |

**Supplementary Table S4. Sequence of primers for Luciferase reporter assay.**

| **MET-promoter-primer** | **Sequence (5’to 3’)** |
| --- | --- |
| MET-promoter -full-left | CTAGCTAGCAGTCAGGCCGCGTTGTTTAT |
| MET-promoter -full-right | CCGCTCGAGATCTGCTCACAAAGCGCTCG |
| MET-promoter –1-left | CTAGCTAGCCTCGCCTCCCAAGCGCCAGG |
| MET-promoter –1-right  MET-promoter –2-left  MET-promoter –2-right  MET-promoter –3-left  MET-promoter –3-right  MET-promoter –4-left  MET-promoter –4-right | CCGCTCGAGATCTGCTCACAAAGCGCTCG  CTAGCTAGCTTCTTACTACATTTTTCTAT  CCGCTCGAGATCTGCTCACAAAGCGCTCG  CTAGCTAGCAGTCAGGCCGCGTTGTTTAT  CCGCTCGAGTAGTTATCATTTCCCAAGTG  CTAGCTAGCTTCTTACTACATTTTTCTAT  CCGCTCGAGTGTCACCCCGGGCTCAGATG |
