# Supplementary figures and images for: CREB5 promotes invasiveness and metastasis in colorectal cancer by directly activating MET
Source: J Exp Clin Cancer Res. 2020 Aug 25;39:168. doi: 10.1186/s13046-020-01673-0 (PMC7446182; doi:10.1186/s13046-020-01673-0)

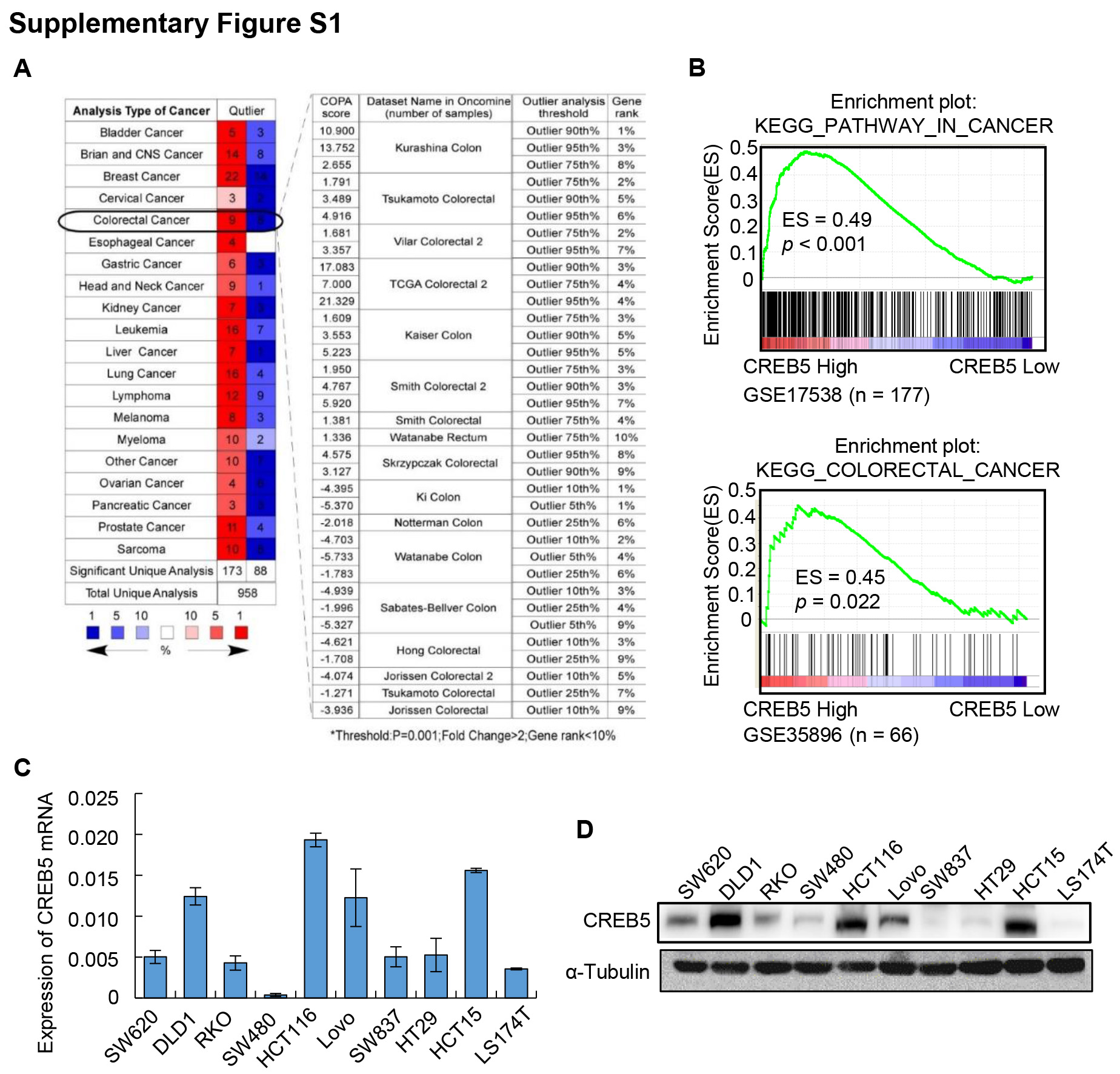

Supplement: Supplementary file 2 — Additional file 2: Figure S1. Bioinformatics analysis of CREB5 expression. The expression of CREB5 in CRC and other malignant tumors was analyzed by Oncomine database. The inclusion criteria were that the difference of CREB5 expression between tumor tissue and normal tissue was more than 2 times, and the arrangement of gene position was less than 10% with P < 0.001. The outliers in the red and blue boxes represent the number of data sets with high and low expression of CREB5, respectively. The right table of (A) represents the COPA score of CREB5 in 17 CRC data sets. (B) The two CRC chips (GSE17538, n = 177, and GSE35896, n = 66) from the public database of GEO was analyzed by GSEA. The plot showed significant enrichment of tumor-related gene set (KEGG_PATHWAY_IN_CANCER) and colorectal cancer-related gene set (KEGG_COLORECTAL_CANCER) in the CREB5 high expression group. (C and D) Real-time PCR and western blotting analysis of CREB5 endogenous expression in indicated CRC cells. [file 13046_2020_1673_MOESM2_ESM.jpg]

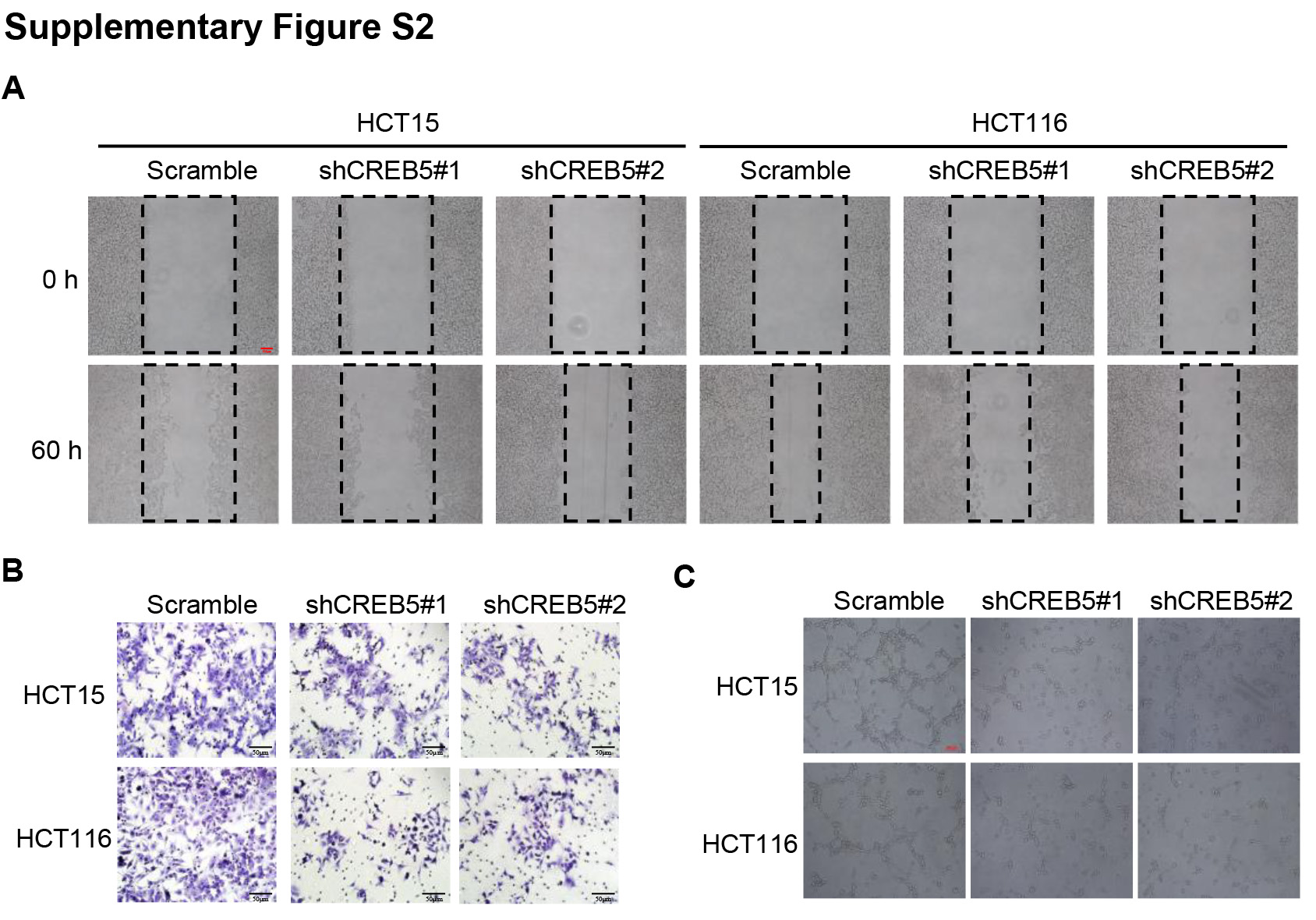

Supplement: Supplementary file 3 — Additional file 3: Figure S2. Representative images of wound-healing assay (A), transwell migration assay (B) and HUVEC tube formation assay (C) with indicated treatment. Scale bars, 100 μm in (A) and (C). 50 μm in (B). [file 13046_2020_1673_MOESM3_ESM.jpg]

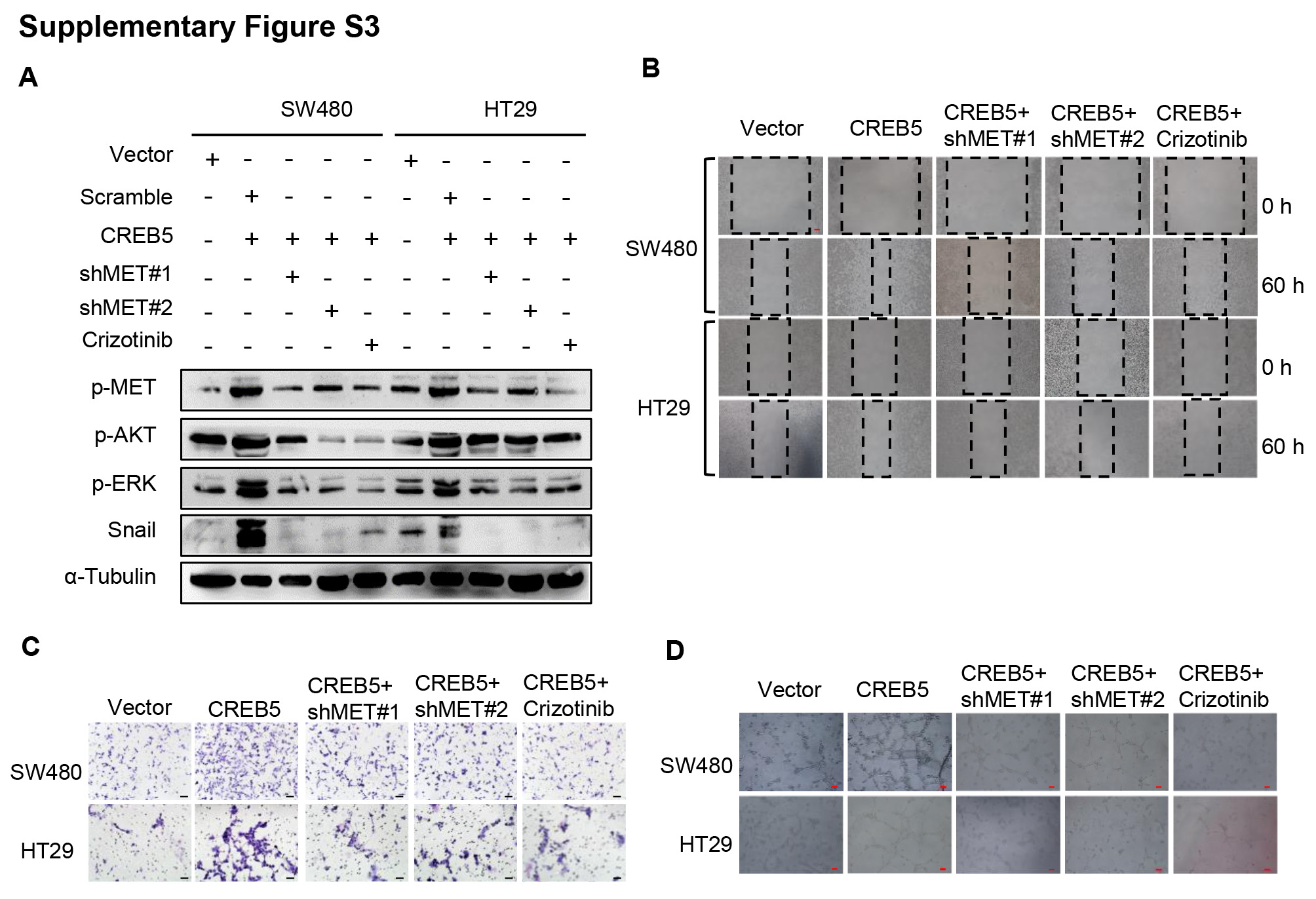

Supplement: Supplementary file 4 — Additional file 4: Figure S3. Two MET shRNAs or inhibitors (Crizotinib) were used in SW480 and HT29 cells overexpressed CREB5, and the expression of MET, p-MET, p-AKT and p-ERK were analyzed by western blotting. ** p < 0.01. Representative images of wound-healing assay (B), transwell migration assay (C) and HUVEC tube formation assay (D) with indicated treatment were shown. Scale bars, 100 μm in (B) and (D). Scale bars, 50 μm in (C). [file 13046_2020_1673_MOESM4_ESM.jpg]
